# Abiotic stress-induced accumulation of raffinose in Arabidopsis leaves is mediated by a single raffinose synthase (*RS5*, At5g40390)

**DOI:** 10.1186/1471-2229-13-218

**Published:** 2013-12-20

**Authors:** Aurélie Egert, Felix Keller, Shaun Peters

**Affiliations:** 1 Institute of Plant Biology, Molecular Plant Physiology Division, University of Zürich, Zollikerstrasse 107, Zürich CH-8008, Switzerland; 2 Institute for Plant Biotechnology, Department of Genetics, Faculty of AgriSciences, Stellenbosch University, Matieland 7602, South Africa; 3 Present address: Molecular and Systems Toxicology, Department of Pharmaceutical Sciences, University of Basel, Klingelbergstrasse 50, Basel CH-4056, Switzerland

**Keywords:** Abiotic stress, Arabidopsis, Galactinol, Raffinose, Raffinose synthase, Seeds, Water-soluble carbohydrates

## Abstract

**Background:**

The sucrosylgalactoside oligosaccharide raffinose (Raf, Suc-Gal_1_) accumulates in Arabidopsis leaves in response to a myriad of abiotic stresses. Whilst galactinol synthases (GolS), the first committed enzyme in Raf biosynthesis are well characterised in Arabidopsis, little is known of the second biosynthetic gene/enzyme raffinose synthase (RS). Conflicting reports suggest the existence of either one or six abiotic stress-inducible RSs (*RS-1* to -*6*) occurring in Arabidopsis. Indirect evidence points to At5g40390 being responsible for low temperature-induced Raf accumulation in Arabidopsis leaves.

**Results:**

By heterologously expressing At5g40390 in *E.coli*, we demonstrate that crude extracts synthesise Raf *in vitro*, contrary to empty vector controls. Using two independent loss-of-function mutants for At5g40390 (*rs 5–1* and *5*–*2*), we confirm that this RS is indeed responsible for Raf accumulation during low temperature-acclimation (4°C), as previously reported. Surprisingly, leaves of mutant plants also fail to accumulate any Raf under diverse abiotic stresses including water-deficit, high salinity, heat shock, and methyl viologen-induced oxidative stress. Correlated to the lack of Raf under these abiotic stress conditions, both mutant plants lack the typical stress-induced RafS activity increase observed in the leaves of wild-type plants.

**Conclusions:**

Collectively our findings point to a single abiotic stress-induced RS isoform (*RS5,* At5g40390) being responsible for Raf biosynthesis in Arabidopsis leaves. However, they do not support a single RS hypothesis since the seeds of both mutant plants still contained Raf, albeit at 0.5-fold lower concentration than seeds from wild-type plants, suggesting the existence of at least one other seed-specific RS. These results also unambiguously discount the existence of six stress-inducible RS isoforms suggested by recent reports.

## Background

Raffinose synthase (RS, EC 2.4.1.82) is an important enzyme involved in the biosynthesis of the raffinose family oligosaccharides (RFOs; Suc-[Gal]_n_, 13 < n ≥ 1) which are α1,6-galactosyl extensions of sucrose (Suc) occurring frequently in higher plants. The first step in their biosynthesis is initiated by galactinol synthase (GolS, EC 2.4.1.123) which catalyses the formation of galactinol (Gol; 1-O-α-D-galactopyranosyl-L-*myo*-inositol), using UDP-galactose (UDP-Gal) and *myo*-inositol (Ino) as substrates. The second step involves RS which transfers the galactosyl moiety from Gol to the C_6_ position of the glucose (Glc) moiety in Suc, forming an α1,6-galactosidic linkage to yield the trisaccharide raffinose (Raf, Suc-Gal_1_). In a third step, stachyose synthase (SS, EC 2.4.1.67) transfers the galactosyl moiety from Gol to the C_6_ position of the Gal moiety in Raf to yield the tetrasaccharide stachyose (Sta, Suc-Gal_2_). Biosynthesis of higher RFO oligomers occurs *via* a Gol-independent biosynthetic pathway. In *Ajuga reptans*, galactan:galactan galactosyl transferase (GGT) utilizes RFOs as both galactosyl donors and acceptors during chain elongation, facilitating the synthesis of higher RFO oligomers (up to Suc-(Gal)_13_; [1-4]). GGT activity has also been reported to occur in leaves of *Coleus blumei*[5].

Plant RSs are generally poorly characterised and putative gene sequences have been reported for only a few plants, including pea, cucumber, maize, grape, rice and Arabidopsis [6]. To date, the only extensive biochemical characterisation of a RS has been reported from pea seeds [7], cucumber [8] and to a lesser degree from rice [9]. Arabidopsis is reported to contain six putative abiotic stress-inducible *RS* genes [*RS1-6*, 10]. However, we have recently demonstrated that *RS2* (At3g57520/*ATSIP2*), contrary to being reported as an abiotic stress-inducible RS [10-12], is in fact a *bona fide* alkaline α-galactosidase (α-Gal, [13]). Conversely, *RS5* (At5g40390) is annotated as being similar to the α-Gal *ATSIP1,* but evidence suggests that it is a RS [14]. Based on sequence similarity to the known pea RS, *RS5* has also been suggested to be the only RS isoform in Arabidopsis [6]. In the absence of complete functional characterisation of the six putative RSs, their actual number in Arabidopsis is a matter of speculation.

In Arabidopsis vegetative tissues (leaves and roots), the only RFO to accumulate is Raf, occurring exclusively during exposure to a myriad of abiotic stresses [10,15]. In mature Arabidopsis seeds, however, both Raf and Sta accumulate [16-18]. Little is known of RS or their contribution to RFO physiology in Arabidopsis, despite comprehensive identification and characterisation of the 10 *GolS* genes in this plant [10,15]. Using a reverse-genetic approach, some indirect evidence points to *RS5* being a true RS [14]. In that study, a single T-DNA loss-of-function mutant was shown to lack Raf in the leaves after 14 d of cold treatment at 4°C, suggesting *RS5* to be involved in cold stress-induced Raf accumulation. The gene was neither functionally identified nor were its gene expression, enzyme activity or temporal Raf accumulation during the 14 d treatment determined. However, in a separate study significant increases and decreases of *RS5* transcripts were reported to occur in Arabidopsis leaves subjected to cold acclimation and deacclimation, respectively [19]. High salinity (150 mM NaCl) and abscisic acid (ABA, 25 μM) treatments would also appear to result in an increase of *RS5* transcripts [20]. In that study, although transcripts increased under both treatments, Raf only accumulated under high salinity and no loss-of-function mutant for *RS5* was analysed.

In the present work, we further characterised *RS5* as a *bona fide* RS by heterologous expression and functional identification in *E. coli*. We also characterised *in vivo* RS activity and Raf accumulation against two additional T-DNA loss-of-function mutants (*rs 5–1* and *5-2*) following (i) cold-stress (4°C), (ii) water deficit, (iii) high salinity (100 mM NaCl), (iv) methyl viologen (MV)-induced oxidative stress (25 μM), and (v) heat shock (30°C). Surprisingly, the RS activity of *RS5* encompassed all of these different abiotic stresses with both mutants showing no RS activity and failing to accumulate any Raf in the leaves. Our findings firmly place *RS5* (At5g40390) as the only RS in Arabidopsis leaves orchestrating abiotic stress-induced Raf accumulation.

## Results

### Crude extracts from *E. coli* heterologously expressing RS5 produce Raf

**Figure 1:**
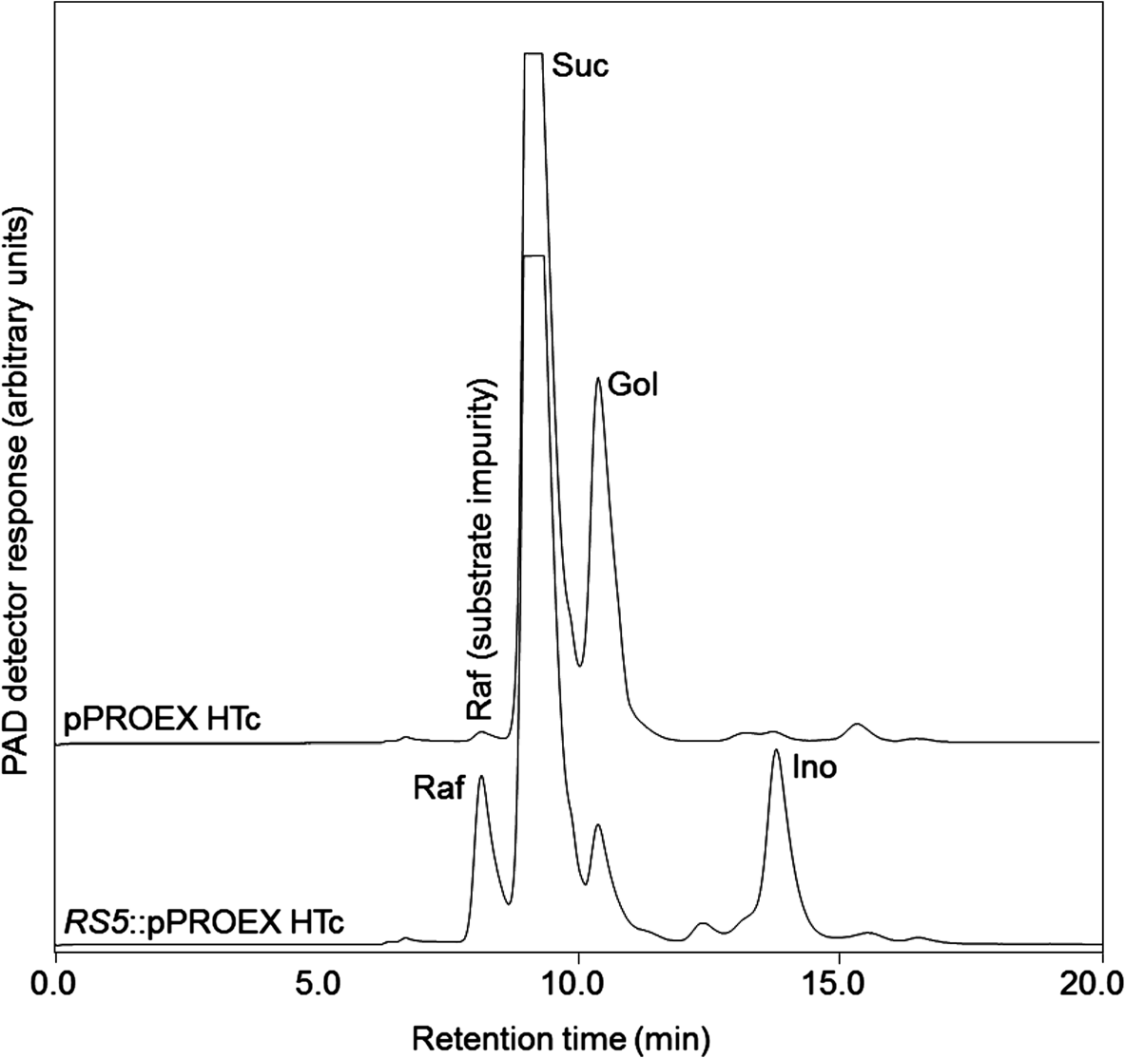
**RS activity in crude extracts from *E. coli *transformed with *RS5*:pPROExHTc (lower chromatogram).** Crude extracts were incubated with 100 mM Suc and 10 mM Gol at pH 7.5 for 1 h. The empty vector control, pPROEx HTc, did not show any RS activity (upper chromatogram). Raf, raffinose (7.2 min); Suc, sucrose (8.8 min); Gol, galactinol (11.2 min); Ino, *myo-*inositol (13.9 min). The Raf biosynthetic reaction yields Ino consequent to the transfer of a galactose moiety from Gol to Suc.

**Figure 2:**
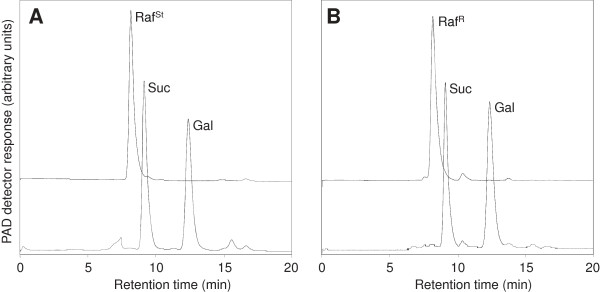
**HPLC-PAD chromatograms confirming the identity of Raf (Raf**^**R**^**) produced by recombinant RS5. A**, Fractions representing 40 nmol of a commercial Raf standard (Raf^St^) were collected after separation by HPLC, hydrolysed with a fungal (*Aspergillus niger*) acid α-Gal and rechromatographed. **B**, Fractions representing Raf^R^ were collected from RS enzyme activity assays after separation by HPLC, hydrolysed as above and rechromatographed. Raf, raffinose (8.2 min); Suc, sucrose (9.2 min); Gal, galactose (12.4 min).

The *RS5* cDNA was cloned into the pPROEX HTc vector and heterologously expressed in *E. coli* (BL21, codon plus). When incubated with 50 mM Suc and 5 mM Gol at pH 7.5, crude extracts from *E. coli* cultures induced for recombinant protein expression with isopropyl β-D-1-thiogalactopyranoside (IPTG; 1 mM, 37°C, 4 h) were clearly able to synthesise a compound which eluted at the same retention time as a commercial Raf standard (Figure 1, lower chromatogram). This was contrary to crude extracts from *E. coli* cultures transformed with the pPROEX HTc vector (Figure 1, upper chromatogram). The identity of the putative Raf produced by the recombinant *RS5* (Raf^R^, Figure 2B) was confirmed by collecting the corresponding fractions after HPLC separation and hydrolysing them with an acid α-Gal. Identical to a commercial Raf standard (Raf^St^, Figure 2A), Raf^R^ was hydrolysed to Suc and Gol in a 1:1 mole ratio (Figures 2A and B). Crude extracts containing recombinant RS5 were also tested for alkaline α-Gal activity in the presence of 50 mM Raf at pH 7.5. No such activity was detected (Additional file 1: Figure S1), precluding *RS5* as an alkaline α-Gal.

### Seeds from *rs 5–1* and *5*–*2* plants show reduced Raf concentrations

**Figure 3:**
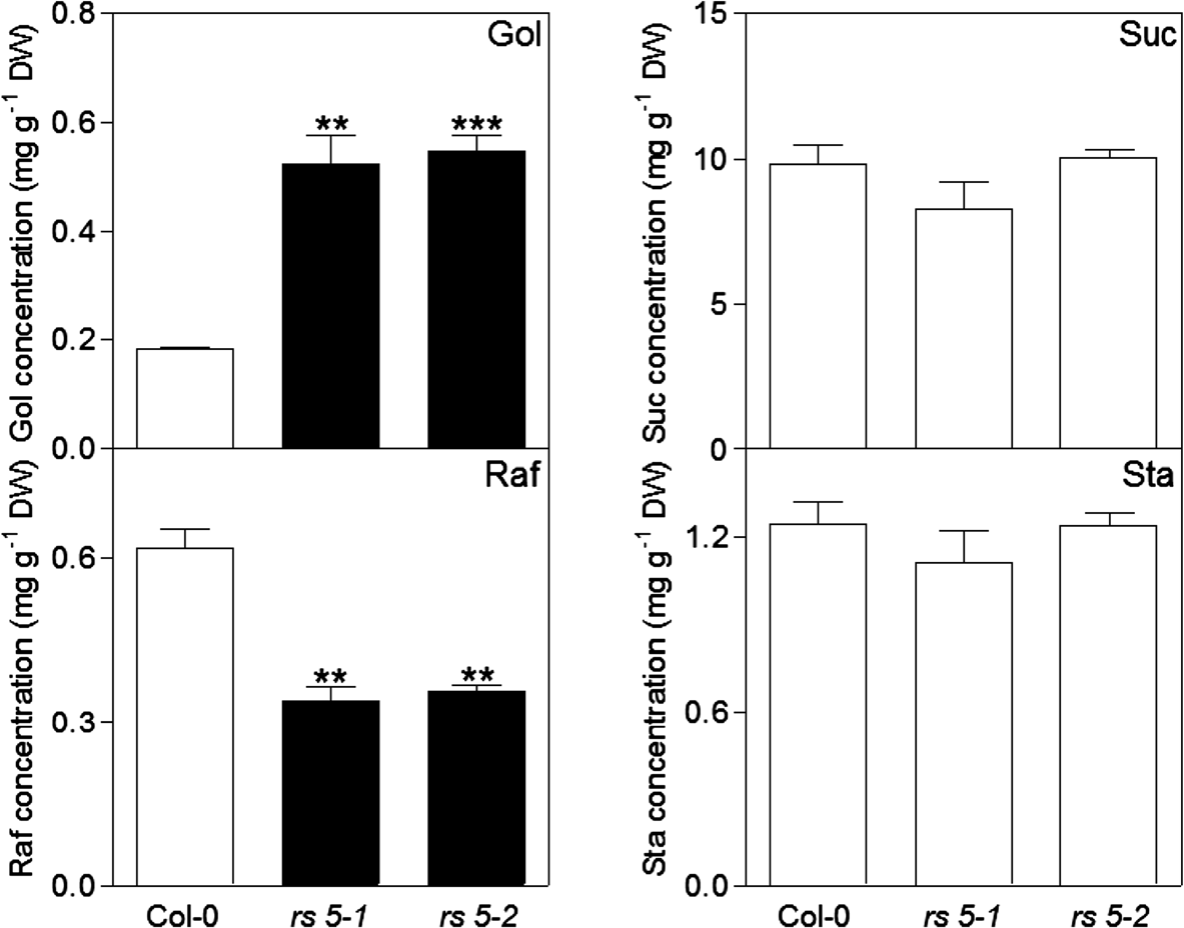
**Water-soluble carbohydrates in seeds of Arabidopsis wild-type (Col-0) and *rs 5–1 *and *5*–*2 *mutant plants.** Gol, galactinol; Suc, sucrose; Raf, raffinose; Sta, stachyose. Values are the means ± SE of three independent replicates. Statistical significance of a two-tailed *t*-test is represented by stars (Gol, **p < 0.002, ***and p < 0.0002; Raf, **p < 0.003 and 0.002 for *rs 5–1* and *5*–*2*, respectively).

Water-soluble carbohydrates (WSCs) were extracted from the seeds of mature wild-type and the *rs 5–1* and *5*–*2* mutants, and analysed by HPLC-PAD (Figure 3). The only RFOs present in wild-type seeds were Sta (1.24 ± 0.076 mg g^-1^ DW) and Raf [(0.62 ± 0.034 mg g^-1^ DW). These RFOs were also present in seeds from *rs 5–1* and *5*–*2* [Sta (1.11 ± 0.11 and 1.24 ± 0.04 mg g^-1^ DW, respectively)] and Raf (0.34 ± 0.04 and 0.35 ± 0.027 mg g^-1^ DW, respectively)]. Raf concentrations were reduced by about half in the seeds of both *RS5* mutants. Gol hyper-accumulated almost 3-fold in seeds from the *RS5* mutants, occurring at concentrations of 0.52 ± 0.051 and 0.54 ± 0.026 mg g^-1^ DW, respectively. Like the Sta concentrations, those of Suc were about equal in all seed types (9.84 ± 0.066, 8.32 ± 0.92 and 10.05 ± 0.27 mg g^-1^ DW for wild-type, *rs 5–1* and *5*–*2*, respectively).

### Crude extracts from the leaves of *rs 5–1* and *5*–*2* mutants are deficient in RS activity under multiple abiotic stresses

To further characterise *RS5* loss-of-function, we reproduced the cold stress experiment previously described [14], using the two additional T-DNA insertion mutants, *rs 5–1* and *5*–*2*. Desalted crude enzyme extracts were incubated with either 5 mM UDP-Gal and 50 mM Ino (GolS activity) or 100 mM Suc and 10 mM Gol (RS activity) at pH 7.5.

**Figure 4:**
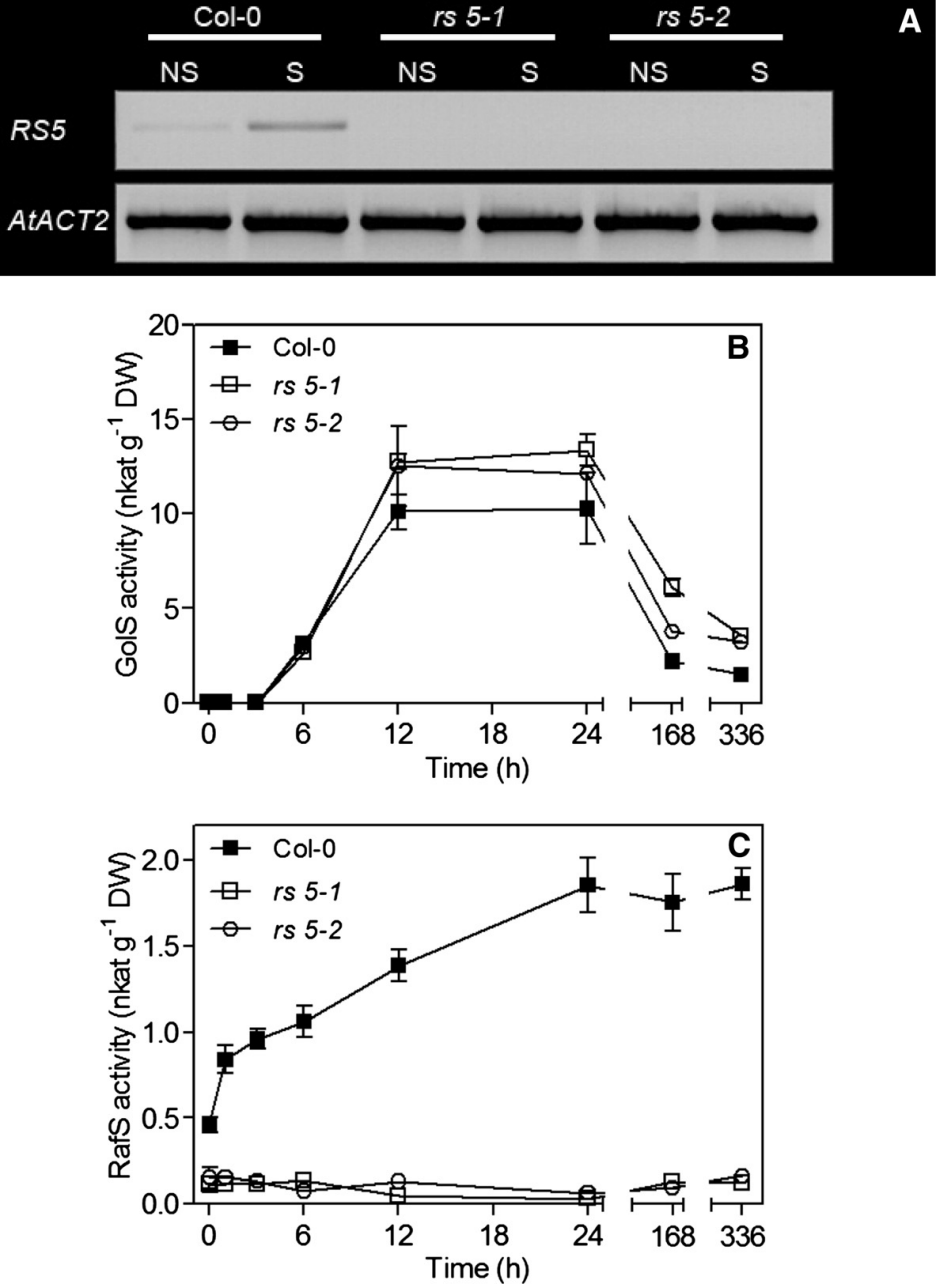
**Analyses of 4°C cold stressed-leaves from wild-type (Col-0) and *RS5 *mutants. A**, Semi-quantitative PCR of *RS5* transcript abundance in the leaves of Col-0, *rs 5–1* and *5*–*2* mutant plants stressed for 24 h. The *ACTIN2* gene *(ACT2, At3g18780)* was used as a constitutively expressed control. **B**, GolS activity in the leaves of Col-0, *rs 5–1* and *5*–*2* plants stressed for 14 d. **C,** RS activity in the leaves of Col-0, *rs 5–1* and *5*–*2* plants stressed for 14 d.

While wild-type plants showed a clear transcriptional induction of *RS5* after 24 h of cold stress at 4°C, the leaves of the *RS5* mutants were transcript deficient (Figure 4A). Further, when GolS activity was measured under these stress conditions, a clear and comparable increase over the first 12 h of cold stress was measured in the leaves of both wild-type and the *RS5* mutant plants. Thereafter, GolS activity remained constant until 24 h of stress and declined steadily at 7 d and 14 d of stress (Figure 4B). RS activity in the leaves of wild-type plants increased linearly over the stress period from about 0.46 ± 0.046 to 1.86 ± 0.138 nkat g^-1^ DW after 14 d of stress at 4°C. Conversely, the leaves of both *rs 5–1 and 5–2* plants showed only trace activities (0.11 ± 0.061 and 0.15 ± 0.044 nkat g^-1^ DW, respectively) which remained unchanged over the duration of the stress (Figure 4C).

Under a battery of abiotic stresses, including water deficit, high salinity (100 mM NaCl), MV-induced oxidative stress, and heat shock (30°C), RS activity in the leaves of wild-type plants increased (Table 1). Conversely, either trace activities or no RS activity was detected in the leaves of the *rs 5–1 and 5–2* mutants under the same stress conditions.

**Table 1:** **RS activities in leaves of wild-type (Col-0) and *RS5 *** **mutants subjected to various abiotic stresses**

	**4°C**		**WD**		**NaCl**		**MV**		**30°C**	
	**NS**	**S**	**NS**	**S**	**NS**	**S**	**NS**	**S**	**NS**	**S**
Col-0	0.46 ± 0.046	**1.86 ± 0.138**	0.55 ± 0.014	**1.17 ± 0.065**	0.18 ± 0.005	**0.34 ± 0.002**	0.22 ± 0.004	**0.33 ± 0.006**	0.24 ± 0.004	**0.33 ± 0.002**
*rs 5-1*	0.15 ± 0.044	0.16 ± 0.028	0.16 ± 0.006	0.14 ± 0.002	n.d.	n.d.	n.d.	n.d.	n.d.	n.d.
*rs 5-2*	0.11 ± 0.061	0.12 ± 0.025	0.17 ± 0.009	0.16 ± 0.002	n.d.	n.d.	n.d.	n.d.	n.d.	n.d.

Assays were conducted with desalted leaf crude extracts incubated with 100 mM Suc and 10 mM Gol at pH 7.5 for 1 h. NS, non-stressed; S, stressed (as per Materials and Methods); 4°C, cold-stressed; WD, water deficit-stressed; NaCl, salt-stressed; MV, methyl viologen-stressed; 30°C, heat-stressed. Values in bold represent increased RS activities in stressed as compared to non-stressed leaves. Values are the means ± SE of three independent replicates and represent RS activities expressed in nkat g^-1^ DW.

### The leaves of *rs 5–1 and 5–2* mutants are deficient in Raf under multiple abiotic stresses

HPLC-PAD analyses of total leaf WSCs from Col-0 and *RS5* mutant plants demonstrated that Raf accumulated in Col-0 leaves under conditions of cold stress, water deficit, high salinity, MV-induced oxidative stress and heat shock. This Raf increase was variable, ranging from a 9-fold increase (about 3 mg g^-1^ DW, 3 h, 25 μM MV) to a 121-fold increase (40 mg g^-1^ DW, 14 d, 4°C). Conversely, the leaves of *rs 5–1 and 5–2* mutants were completely deficient of Raf under the same stress conditions (Table 2).

**Table 2:** **Raf concentrations in leaves of wild-type (Col-0) and *RS5 *** **mutants subjected to various abiotic stresses**

	**NS**	**4°C**	**WD**	**NaCl**	**MV**	**30°C**
Col-0	0.33 ± 0.012	**40.00 ± 1.488**	**11.00 ± 0.221**	**17.00 ± 1.577**	**3.03 ± 0.603**	**10.15 ± 0.622**
*rs 5-1*	n.d.	n.d.	n.d.	n.d.	n.d.	n.d.
*rs 5-2*	n.d.	n.d.	n.d.	n.d.	n.d.	n.d.

NS, non-stressed; S, stressed (as per Materials and Methods); 4°C, cold-stressed; WD, water deficit-stressed; NaCl, salt-stressed; MV, methyl viologen-stressed; 30°C, heat-stressed. Values in bold represent increased Raf in stressed as compared to non-stressed leaves. Values are the means ± SE of three independent replicates and represent Raf concentrations expressed as mg g^-1^ DW.

## Discussion

### *RS5* is not the only RS gene in Arabidopsis

Recent reports provide conflicting evidence suggesting the existence of either a single [*RS5*; 6] or six [*RS-1* to -*6*; 10] RS isoform/s occurring in Arabidopsis. We have recently discounted *RS2* (At3g57520/*ATSIP2*) as a RS, based on functional expression of the cDNA [13]. In that study, we demonstrated that *ATSIP2*, when expressed in *Sf9* insect cells, lacked any RS activity, but rather showed a distinct hydrolase activity with a preference for Raf as a substrate, identifying ATSIP2 as a genuine alkaline α-Gal. By heterologously expressing the *RS5* cDNA in *E. coli* we could demonstrate, *in vitro*, that crude extracts containing recombinant RS5 synthesised Raf and presented no α-Gal activity (on Raf, Additional file 1: Figure S1), confirming that *RS5* encodes for a genuine RS.

We hypothesised that if *RS5* was the sole Arabidopsis RS as previously suggested [6] then mature seeds from the *rs 5–1* and *5–2* mutants would show complete ablation of Raf (and possibly Sta) accumulation. It is well reported that, apart from Suc, mature Arabidopsis seeds accumulate substantial quantities of RFOs [mainly Raf and Sta; 16, 17, 18]. Seeds of both *rs 5–1 and 5–2* mutants showed reduced Raf concentrations to about 50% of seeds from wild-type plants. This still represented concentrations of about 0.35 mg g^-1^ DW, for *rs 5–1* and *5–2* seeds (Figure 3). The concentrations of the other major seed WSCs, Sta and Suc, were comparable between the mutants and those of wild-type plants (Figure 3). These observations argue for the existence of at least one other, as yet unidentified, RS in Arabidopsis which is responsible for Raf accumulation during seed development. Importantly, we could allocate an additional physiological role to *RS5* in Raf accumulation during seed development, apart from its already reported role in Raf accumulation in Arabidopsis leaves during cold stress [14].

### *RS5* is the only RS gene responsible for Raf accumulation in leaves during abiotic stress

To further investigate the existence of other putative *RS* genes *in vivo*, 5-week old soil-grown wild-type and mutant plants were subjected to a battery of abiotic stresses (cold stress, water deficit, high salinity, heat shock and MV-induced oxidative stress). All of these stress conditions result in the accumulation of Raf in the leaves of Arabidopsis and have been shown to differentially up-regulate various GolS isoforms in leaves [10,14,15,21]. Surprisingly, while wild-type plants accumulated Raf in the leaves under all conditions tested, both *RS5* mutants failed to do so in their leaves (Table 2) suggesting that At5g40390 is the only RS, in leaves, responsible for abiotic stress-induced Raf accumulation.

Further evidence was obtained by measuring the total extractable RS activity in the leaves of wild-type and *RS5* mutant plants during exposure to the stresses stated above. Under all conditions, an increase in RS activity was measured in leaves of stressed wild-type plants, while leaves of *rs 5–1 and 5–2* mutants presented either trace (cold stress) or no measurable RS activities (Table 1). During our experimentation, we observed distinct variations in RS activity between independent wild-type control plants (unstressed) despite the controlled growth conditions described. The reason for this finding is unclear, but we propose that it may be due to subtle physiological differences between the plants used in these independent experiments.

The ablation of stress-induced RS activity in the *rs 5–1 and 5–2* mutants is astonishing, particularly given that six of the 10 *GolS* genes in Arabidopsis have been implicated in abiotic stress-related RFO metabolism [10,15]. Furthermore, six putative abiotic stress inducible RS genes have recently been reported in Arabidopsis [designated *RS-1* to -*6*, 10]. In the present study using a reverse genetic approach, we describe *RS5* (At5g40390) as being the sole abiotic stress-induced RS in Arabidopsis leaves. Hence, the putative *RS-1*, -*3*, *-4* and -*6* (At1g55740, At4g01265, At4g01970 and At5g20250, respectively) may be precluded as abiotic stress-inducible RSs.

As a consequence of *RS5* loss-of-function, the leaves of *rs 5–1 and 5–2* mutant plants subjected to abiotic stresses hyper-accumulated Gol but not Raf. The GABI-KAT T-DNA insertion mutant previously reported also hyper-accumulated Gol in the leaves after cold stress [4°C, 14 d; 15]. The role of Gol as a legitimate stress protectant has been largely overlooked, perhaps because the occurrence of Gol is always linked to the presence of Raf (the most widely reported WSC with protective functions). Our findings clearly demonstrate that *RS5* loss-of-function mutants provide a Raf-free biological system during exposure to abiotic stress, potentially providing a means to analyse a functional role for Gol in stress protection. Experiments are currently underway with a particular focus on dissecting the possible protective effects that Gol hyper-accumulation may facilitate in the absence of Raf.

## Conclusions

Using a reverse genetic approach *RS5* (At5g40390) has previously only been characterised as a low temperature-induced RS contributing to Raf accumulation in Arabidopsis leaves. Using two additional loss-of-function mutants we could demonstrate further that RS5 (i) is the sole RS responsible for Raf accumulation in Arabidopsis leaves exposed to water deficit, high salinity, MV-induced oxidative stress and heat shock and, (ii) also functions in Raf accumulation in Arabidopsis seeds. Collectively, this work unambiguously demonstrates *RS5* is the only RS responsible for Raf accumulation in Arabidopsis leaves during abiotic stress and precludes the existence of five other putative abiotic stress-inducible RSs recently described on the basis of sequence homology.

## Methods

### Plant material and abiotic stress treatments

The *RS5* mutants (*rs 5–1* and 5–*2*) in the Col-0 background were obtained from the SALK collection (SALK_049583 and _085989, respectively). The mutants carry a T-DNA insert in the second intron of At5g40390. Homozygous mutants were identified by PCR using two different primer pairs: the At5g40390 wild-type allele was amplified with the primers *rs 5–1* (fwd 5^′^ CTCTTCTTGAAGGCTCCTTCC, rev 5^′^ ATGACATCAACTTTAACGCCG) and *rs 5–2* (fwd 5^′^ATGGAACTCAGCACAAGGATG, rev 5^′^TTATTGAAATCCTCACACC). The mutant allele was amplified with the SALK Lb1.3 primer (5^′^TTTTGCCGATTTCGGAAC) and either the *rs 5–1* or *5*–*2* reverse primer, respectively. Wild-type plants used in this study represented a pool (3) of individual plants which genotyped as wild-type in the screen described above.

Following seed stratification (48 h, 4°C), Arabidopsis plants were propagated in soil (Einheitserde, type ED73, Gebr. Patzer GmbH & Co. KG, Schopfheim, Germany) in a controlled environment chamber (8 h light, 30 μmol photons m^-2^ s^-1^, 22°C, 16 h dark, 60% RH). Five-week-old plants were used for abiotic stress treatments as follows. Experiments were conducted twice, using 4 pools of 6 plants (24 plants) per experiment.

**(i) *cold stress***: plants were transferred into an acclimation chamber with identical settings to those listed above but with a constant temperature of 4°C, for a period of 14 d; **(ii) *high salinity*** was imposed by irrigating soil with 150 ml of NaCl solution (25 mM, after 24 h 50 mM and 24 h thereafter 100 mM); **(iii) *water deficit***: soil was passed through a sieve (0.5 x 0.5 cm mesh size) to remove large detritus and 60 g was weighed into pots prior to seed sowing. The pots were sub-irrigated twice weekly until plants reached the desired growth stage (5-week-old). Water deficit was subsequently imposed by withholding water until leaves showed the first visible signs of leaf wilting, typically occurring between 7 and 9 d under our growth conditions, **(iv) *oxidative stress*** was imposed as previously described [10], with minor modifications. Leaves were liberally sprayed with 25 μM MV (paraquat) followed by a 3 h exposure to high light-intensity (700 μmol photons m^-2^ s^-1^); **(v) *heat shock*** was imposed by transferring plants to 30°C for 6 h.

### RNA isolation and semiquantitative PCR (sqPCR)

Total RNA was extracted from leaves using the RNeasy mini kit (Qiagen, Hombrechtikon, Switzerland), following the manufacturer’s instructions. The cDNA template for sqPCR were obtained by reverse transcription of 1 μg total RNA with an oligo (dT_15_) primer and the M-MLV reverse transcriptase (Promega AG, Dübendorf, Switzerland) following the manufacturer’s instructions. The sqPCR was carried out in 50 μl containing 5 μl cDNA, 0.5 mM of each dNTP, and 0.5 μmol of each primer, 1X PCR buffer and 1.25 U GoTaq DNA polymerase (Promega), at a primer annealing temperature of 56°C for 24 cycles. The number of cycles chosen for the sqPCR was determined to occur in the linear range of the constitutively expressed *ACTIN2* gene *(ACT2,* At3g18780). The following primer pairs were used to amplify a 1Kb fragment of the corresponding cDNAs *ACTIN2*: *ACT2*_*fwd*_ 5´ATGGCTGAGGCTGATGATAT, *ACT2*_*rev*_ 5′TTAGAAACATTTTCTGTGAACGAT; *RS5*: *RS5*_*fwd*_ 5^′^ ATGGCTTCGCCGTGTTTGACC and *RS5*_*rev*_ 5′ CGGAGCTTCAGGACGGAGAC.

### Heterologous expression of At5g40390 in *E. coli*

Total RNA was isolated from the leaves of cold-stressed (4°C, 14 d) Col-0 Arabidopsis plants using the Plant RNeasy kit (Qiagen AG, Hombrechtikon, Switzerland). The cDNA template for high fidelity PCR of the At5g40390 open reading frame (ORF) was obtained by reverse transcribing 1 μg total RNA with an oligo (dT_15_) primer and M-MLV (H^–^) reverse transcriptase (Promega AG, Dübendorf, Switzerland) following the manufacturer’s protocol. The high fidelity PCR was conducted with 2 μl first strand cDNA using the Expand High Fidelity PCR kit (Roche) following the manufacturer’s instructions. The primer pair amplified the entire 2.48 kB ORF of At5g40390 (R*S5*_*fwd*_ 5^′^ ATGGCTTCGCCGTGTTTGACC and *RS5*_*rev*_ 5′ CTAAAACAAATACTGAATAGAAGACAAACC). The resultant amplicon was cloned into the pGEMT-Easy vector (Promega) and subcloned into the pPROEx HTc vector (Invitrogen) using the *Not*I restriction endonuclease. This construct (*RS5*::pPROExHTc) was transformed *via* standard heat shock procedure, into *E. coli* (BL21 Codon Plus, Stratagene).

Induction of recombinant RS5 expression and crude extract preparations were conducted as previously described [22]. Aliquots (25 μl) of crude extracts were assayed for RS activity in a 50 μl final volume containing 25 μl assay buffer (50 mM HEPES-KOH, pH 7.5, 100 mM Suc, 10 mM Gol). Assays were incubated for 1 h at 30°C and subsequently desalted and analysed by HPLC-PAD as previously described [4,13,22,23]. Control reactions represented crude extracts from cell cultures transformed with the empty pPROExHTc vector and processed as outlined above.

To confirm that heterologous RS5 actually produced Raf, fractions of enzyme assay reactions were collected after HPLC separation and digested with a fungal (*Aspergillus niger*) acid α-Gal, as previously described [22]. Further, to confirm that RS5 did not display an alkaline α-Gal activity, crude extracts were incubated in the presence of 50 mM Raf [13].

### Enzyme extractions, GolS and RS activity assays

Freshly harvested Arabidopsis leaf material (200 mg) was ground in 400 μl of chilled extraction buffer [50 mM HEPES/KOH, pH 7.5, 5 mM MgCl_2_, 1 mM EDTA, 20 mM dithiothreitol (DTT), 0.1% (v/v) Triton X-100, 1 mM benzamidine, 1 mM phenylmethylsulphonyl fluoride (PMSF), 50 mM Na-ascorbate, 2% (w/v) polyvinylpyrrolidone (PVP)]. Samples were centrifuged at 12,000 x g (5 min, 4°C). A 200 μl aliquot of supernatant was desalted by gel filtration at 1,400 x g (2 min, 4°C) through 5 ml Sephadex G-25 columns (fine, final bed volume of 3 ml). Columns were pre-equilibrated with assay buffer (50 mM HEPES/KOH, pH 7.5, 2 mM MnCl_2_, 10 mM DTT). Pre-equilibration was performed twice with 2 ml of assay buffer. Aliquots (20 μl) of desalted extract were assayed for GolS activity in a final volume of 40 μl containing 20 μl assay buffer (50 mM HEPES-KOH, pH 7.5, 100 mM Ino, 10 mM UDP-Gal) at 30°C for 20 min. Similarly, RS activity was assayed at 30°C for 60 min with assay buffer (50 mM HEPES-KOH, pH 7.5, 100 mM Suc, 10 mM Gol). To determine the degree of Raf contamination of Suc (substrate impurity), assay buffer was incubated with 20 μl dH_2_0 and processed as described above. Samples were desalted and analysed by HPLC-PAD as previously described [4,22,23]. Enzyme activities were expressed as a measure of dry weight.

### WSC extraction

WSCs were extracted using an ethanol series, as previously described [4,13,22,23], with minor modifications. Ground, freeze-dried Arabidopsis leaf material (100 mg) from 5-week-old soil-grown plants was flash-frozen in liquid N_2_ and macerated, by hand, using plastic pestle in a 1.5 ml Eppendorf tube. WSCs were extracted twice (per step) in a three-step sequential process, using 1 ml of 80% (v/v) EtOH, 50% (v/v) EtOH, and dH_2_O, respectively. Extractions were conducted at 80°C for 10 min and the tubes centrifuged at 15,000 x g (5 min, 4°C). Samples were desalted and analysed by HPLC-PAD as previously described [4,13,22,23].

### HPLC-PAD analysis

Desalted WSC extracts and enzyme assay reactions were analysed and quantified by HPLC-PAD as previously described [4,13,22,23]. Briefly, a Ca^2+^/Na^+^-moderated ion partitioning carbohydrate column (Benson BC100, BC200 columns, 7.8 × 300 mm; Benson Polymeric, Reno, Nevada, USA) was used to separate carbohydrates. Quantification was done using the Chromeleon v6.4 software package (Dionex) against a series of 5 nmol of standard sugars, the concentration of which corresponded to the linear response range of both chromatographic systems.

## Abbreviations

RS: Raffinose synthase; Gol: Galactinol; Raf: Raffinose; Sta: Stachyose; GGT: Galactan:galactan galactosyl transferase; α-Gal: α-galactosidase; GolS: Galactinol synthase; ATSIP1/ATSIP2: *Arabidopsis thaliana* seed imbibition protein 1/2; MV: Methyl viologen; DW: Dry weight.

## Competing interests

The authors declare that they have no competing interests.

## Authors’ contributions

AE conducted the stress experiments and measurements of (i) water-soluble carbohydrates and (ii) enzyme activities. FK participated in design and co-ordination of the study. SP conceived of the study, its design and co-ordination and undertook (i) the heterologous expression in *E.coli* and (ii) identification of the Arabidopsis mutants. All authors read and approved the final manuscript.

## Supplementary Material

Additional file 1: Figure S1 HPLC-PAD chromatogram testing alkaline α-galactosidase (PDF 183 kb)(α-Gal) activity in crude extracts from *E. coli* transformed with *RS5*::pPROExHTc. Crude extracts were incubated with 50 mM Raf at pH 7.5 for 1 h. Crude extacts (from *Sf*9 insect cells) that heterologously expressed ATSIP2 (At3g57520, Peters et al. 2010) were used as a positive control for α-Gal activity. Neither the empty vector control, pPROExHTc, nor *RS5*::pPROExHTc showed any α-Gal activity. Raf, raffinose (8.2 min); Suc, sucrose (9.4 min); Gal, galactose (12.6 min). Click here for file
