# Supplementary material for: Abiotic stress-induced accumulation of raffinose in Arabidopsis leaves is mediated by a single raffinose synthase (RS5, At5g40390)
Source: BMC Plant Biol. 2013 Dec 20;13:218. doi: 10.1186/1471-2229-13-218 (PMC3878221; doi:10.1186/1471-2229-13-218)
Supplement: Additional file 1: Figure S1 — HPLC-PAD chromatogram testing alkaline α-galactosidase (PDF 183 kb)(α-Gal) activity in crude extracts from E. coli transformed with RS5::pPROExHTc. Crude extracts were incubated with 50 mM Raf at pH 7.5 for 1 h. Crude extacts (from Sf9 insect cells) that heterologously expressed ATSIP2 (At3g57520, Peters et al. 2010) were used as a positive control for α-Gal activity. Neither the empty vector control, pPROExHTc, nor RS5::pPROExHTc showed any α-Gal activity. Raf, raffinose (8.2 min); Suc, sucrose (9.4 min); Gal, galactose (12.6 min). [file 1471-2229-13-218-S1.pdf]

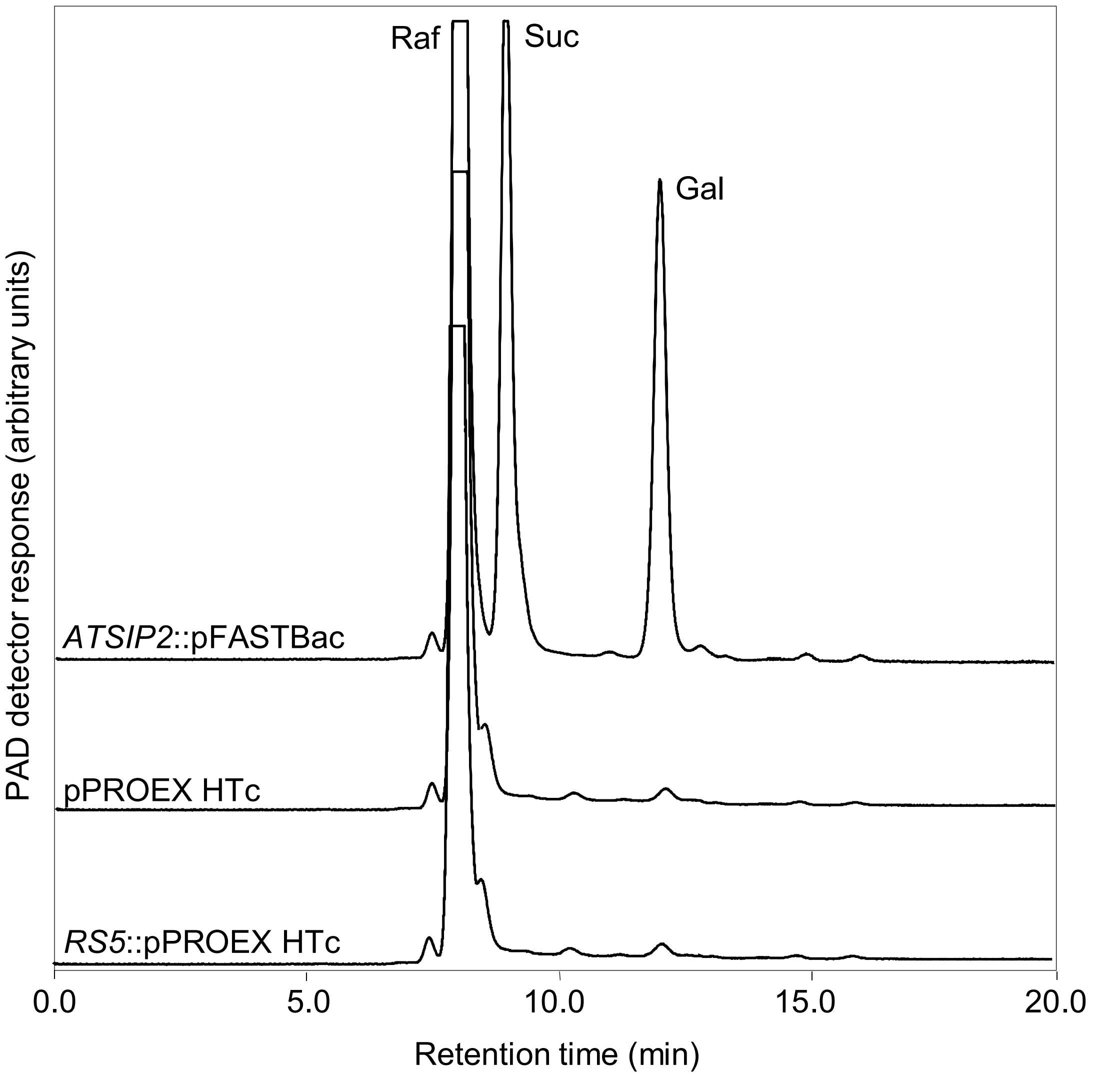

Supplementary Fig. S1 HPLC-PAD chromatogram testing alkaline α-galactosidase (α-Gal) activity in crude extracts from *E. coli* transformed with *RS5*::pPROExHTc. Crude extracts were incubated with 50 mM Raf at pH 7.5 for 1 h. Crude extacts (from *Sf*9 insect cells) that heterologously expressed *ATSIP2* (At3g57520, Peters et al. 2010) were used as a positive control for α-Gal activity. Neither the empty vector control, pPROExHTc, nor *RS5*::pPROExHTc showed any α-Gal activity. Raf, raffinose (8.2 min); Suc, sucrose (9.4 min); Gal, galactose (12.6 min).
